# Maternal Fed Zinc-Deficient Diet: Effects on Relaxin Family Peptides and Oxidant System in the Testis and Liver Tissue of Male Offspring

**DOI:** 10.1007/s12011-024-04113-8

**Published:** 2024-02-26

**Authors:** Hamiyet Kose, Abdullah Sivrikaya, Esma Menevse

**Affiliations:** Department of Medical Biochemistry, Faculty of Medicine, Selcuk University, Konya, Turkey

**Keywords:** Relaxin peptide family, Zinc-deficient diet, Male fertility, Malondialdehyde, Puberty, Glutathione

## Abstract

Today, the studies are limited on roles of insulin-like peptide 3 (INSL3), insulin-like peptide 7 (INSL7), and relaxin family peptide receptor 1 (RXFP1) which are synthesized by the testis. It is aimed to investigate the levels of the sex hormone as testosterone and the family of insulin-like proteins (relaxin family peptides), which are important in the puberty transition, in the testicular and liver tissues of male offspring born to female rats fed a zinc-deficient diet during the pregnancy, and in the changes in lipid peroxidation markers. The study was performed on 40 male offspring. In Group I: Control group, both male offspring and mothers were fed with standard rat chow. In Group II: Zinc deficient diet, both male offspring and mothers were fed a zinc-deficient diet (2.8 mg/kg zinc). In Group III: Normal diet, male offspring fed standard rat chow for 45 days (66th day) after being separated from their mothers with a maternal zinc-deficient diet. In Group IV: Zinc-supplemented diet, offspring fed with zinc supplemented (5 mg/kg/day intraperitoneal zinc sulfate, i.p.) in addition to standard rat chow after being separated from their mothers with maternal zinc deficiency until the termination of the study (66th day). Our study suggests that zinc-supplemented diets play an important role in the changes in INSL3, INSL7, RXFP1, and testosterone levels during spermatogenesis. INSL7, INSL3, and RXFP1 levels were higher in zinc-supplemented group than the zinc-deficient diet group. Liver levels of INSL3, INSL7, and MDA were significantly different in zinc-deficiency diet group than zinc-supplemented group.

## Introduction

Zinc (Zn) is an essential mineral containing more than 2700 enzymes, including antioxidants, metalloenzymes, zinc-binding factors, and zinc transporters that regulate carbohydrate and protein metabolism, DNA and RNA synthesis, cellular replication and differentiation, and hormones [1–3]. Zinc binds to a variety of proteins, thus affecting many biological processes such as cell division, growth, and differentiation as well as structural proteins, enzymatic processes, and transcription factors. The liver is primarily responsible for zinc metabolism [4] and homeostasis [5]. Zinc supplementation can help regulate protein metabolism [6]. Particularly the conversion of testosterone to dihydrotestosterone, as 5α-reductase is a zinc-dependent enzyme [3]. As an antioxidant, zinc also scavenges reactive oxygen species. Fertility and zinc are closely related [7]: seminal plasma acts as a buffer fluid that transfers male gametes to the female genital organs and maintains spermatozoa fertility. Changes in the composition of seminal plasma proteins effect spermatozoa functions [8].

Elucidating the proteins, stimulating factors, and cell–cell interactions that regulate oocyte-spermatozoan will help clarify the regulatory mechanisms of fertilization, and contributes to guide treatments for infertility [9]. Proteins are synthesized by the testis, such as insulin-like peptide 3 (INSL3), insulin-like peptide 7 (INSL7), and relaxin family peptide receptor 1 (RXFP1).

The relaxin peptide family in humans consists of relaxin-1, 2, 3 as well as insulin-like peptides (INSL)-3, 4, 5, and 6 [10]. These peptides are structurally related to insulin consisting of two subunits (chain A and chain B) with disulfide bonds [11]. Relaxin has anti-inflammatory, antioxidant, anti-hypertrophic, anti-apoptotic, angiogenic, wound healing, and vasodilator properties [12]. In addition, this family of peptides exerts antifibrotic effects on the liver [13]. Despite the importance of INSL3 and INSL7 proteins in testicular and bone metabolism [14], their roles in the liver is unclear. These peptides are involved in various diseases and therefore contribute to drug discovery and development [15]. INSL3 and INSL7 interact with relaxin family peptide receptor 3 (RXFP3). Although recent studies on INSL7 have focused on its function in the brain as a neuropeptide, it has been detected in testes and may be a target for spermatozoa [16]. Relaxin detected in seminal plasma may affect both spermatozoa and the acrosome reaction [17]. INSL7 improves Leydig cell viability, differentiation, and sperm motility [16, 18]. This has been shown in the development and function of the male reproductive tract in mice and supports the growth of the prostate and fertilization [18]. INSL7’s specific receptor is RXFP1 [19], which mainly localizes in the astrodome and is expressed in human spermatozoa [20].

The benefits of relaxin treatment in the liver are debatable [21]. Recent findings suggest that INSL3, a hormone produced by Leydig cells, has endocrine, autocrine, and paracrine roles [22]. In males, circulating INSL3 concentration increases in the perinatal period decreases during childhood and peaks in the pubertal period [23].

Its production of INSL3 reflects the differentiation of Leydig cells, the number of cells, and their functional characteristics [24, 25]. Accordingly, INSL3 mediates fetal and postnatal testicular development [22, 26]. Secretion of INSL3 rises during the postnatal “mini-puberty” period [27]; therefore, this family bolsters many functions in spermatozoa, though its precise mechanisms are still unclear.

Testosterone increases as Leydig cells develop, but serum testosterone levels decline with advancing age [28, 29]. In addition to testosterone, INSL3 is essential for testicular descent, which in humans should be completed before birth [25]. However, INSL3 is a more sensitive marker for assessing Leydig cell differentiation, function, and number than testosterone, which is regulated by hypothalamo-pituitary–gonadal hormones [22, 30].

The liver is sexually dimorphic exhibiting major differences in the profile of more than 1000 liver genes related to steroid, lipid, and foreign compound metabolism. The brain, a key regulator of the endocrine system, is thought to regulate sexual dimorphism of the liver [31].

Oxygen radicals are difficult to measure because of their high reactivity, short half-life, and low concentration. To measure tissue destruction due to lipid peroxidation, precursors such as lipid hydroperoxides, conjugated dienes, and peroxy radicals or breakdown products such as lipid hydroperoxides, alkanes, and aldehydes are preferred. Its interaction with DNA and proteins has often been referred to as potentially mutagenic and atherogenic. Malondialdehyde (MDA) is one of these breakdown products [32, 33]. Lipid peroxidation, which starts in plasma and organelle membranes with an increase in free radicals, plays an important role in liver pathogenesis [34, 35]. MDA affects ion exchange through membranes, leading to adverse effects such as ion permeability, alterations in enzyme activity, and cross-linking of membrane compounds [36]. Although low zinc levels increase oxidative damage in the testes, zinc supplementation can reverse these effects and protect against oxidative stress [37, 38].

Given the critical role of zinc during puberty, we aimed to investigate the levels of sex hormones such as testosterone and the family of insulin-like proteins (relaxin family peptides) as well as lipid peroxidation markers in the testicular tissues of male offspring born to female rats fed a zinc-deficient diet during pregnancy. In addition, as 90% of sex-specific liver genes depend on sexually dimorphic GH secretion patterns, we measured the levels of insulin-like proteins (relaxin family peptides) and changes in oxidant markers in liver tissue after administering a zinc-deficient diet, a normal diet, and zinc-supplemented diets to male offspring born with a maternal zinc-deficient diet.

## Materials and Methods

The present study was performed with the decision of Selcuk University Experimental Medicine Research and Application Centre Animal Experiments Ethics Committee (Decision no: 2019–19). The study was supported by Selcuk University Scientific Research Projects Coordination Office (Project no: 19202040). In the study, 40 Wistar Albino male pubertal rats weighing 250–300 g were obtained from Selcuk University Experimental Medicine Research and Application Center. Rats were kept under standard laboratory conditions (21 ± 1 °C, humidity 55 ± 5%) for 12 h in a light/dark environment and fed with normal rat chow. To obtain male offspring, 15 adult female rats (for experimental groups) and 5 adult male rats were randomly selected. After a certain period of time (10 days), male rats were removed from the cages, and females were checked twice a day (8:00 a.m. and 5:00 p.m.) for pregnancy. Pregnancy was determined by the presence of vaginal plaque (embryonic day 0).

### Experimental Groups

Pregnancy detected female rats were fed with a zinc deficient (2.8 mg/kg zinc) (Table 1) diet from the initial of the pregnancy until the 21st day after giving birth. On day 21, they separated from their male pups. The offspring in the groups were all male pups with no sexual experience. Male offspring (21 days old) were divided into 4 groups as follows:

- Group I: Control group (*n* = 10). Both male offspring and mothers (during their pregnancy and 21 days after giving birth) were fed with standard rat chow (Table 1).
- Group II: Zinc deficient diet group (*n* = 10). The offspring were fed a zinc-deficient diet (2.8 mg/kg zinc) after being separated from their mothers until the study was terminated (on the day 66th) (Table 2).
- Group III: Normal diet group (*n* = 10). Male offspring fed standard rat chow for 45 days (66th day) after being separated from their mothers with a maternal zinc-deficient diet.
- Group IV: Zinc-supplemented diet group (*n* = 10). Offspring fed with zinc supplemented (5 mg/kg/day intraperitoneal zinc sulfate, i.p.) in addition to standard rat chow after being separated from their mothers with maternal zinc deficiency until the termination of the study (66th day).

**Table 1:** Content of some trace elements and minerals in standard rat diet

Content	Value	Unit
Zinc	95.18	mg/kg
Copper	2.81	mg/kg
Magnesium	2.220	mg/kg
Calcium	7.012	mg/kg
İron	3.484	mg/kg
Sodium	2.128	mg/kg
Potassium	8.797	mg/kg
Sulfur	1.141	mg/kg
Manganese	95.06	mg/kg
Cobalt	0.34	mg/kg
Molybdenum	1.10	mg/kg
İodine	1.66	mg/kg
Selenium	0.25	mg/kg
Phosphorus	5.014	mg/kg

Energy content was 2091 (65%) kcal/kg for carbohydrates, 367 (11%) kcal/kg for fat, and 768 (%24) kcal/kg for protein. Vitamin mix, the vitamin mix of feed given to experimental animals contains vitamins A, B1, B2, B6, B12, D3, E, K and folic acid, nicotinamide, choline, chloride and D-biotin

**Table 2:** Content of the zinc-deficient diet

Nutritional contents	Ratio (%)
Zinc (mg/kg)	2.8
Copper (mg/kg)	0.25
Magnesium (mg/kg)	2.128
Calcium (mg/kg)	7.012
İron (mg/kg)	3.484
Sodium (mg/kg)	2.220
Glucose	56.3%
Corn oil	8%
Soy protein	30%
Vitamin blend	1.0%
Salt mixture	4%
DL-methionine	0.7%

### Surgical Procedures

At 24 h after termination of the study, testicular and liver tissue samples were obtained from the rats sacrificed under anesthesia using the combination of Ketamine hydrochloride (60 mg/kg, Park-Davis) and Xylazine (5 mg/kg, Bayer).

### Biochemical Analysis

For biochemical analysis, the wet weights of the tissues were recorded, then 10% homogenate was prepared with pH 7.4 PBS (0.01 M, pH = 7.4, Sigma P-4417, Germany) and homogenized in Misonix Microscan ultrasonic tissue shredder at + 4 °C. The samples were centrifuged at 3000 rpm for 15 min in a + 4 °C (Allegra X-30, Beckman Coulter, Turkey). Then, the supernatants were separated and stored in Eppendorf tubes. INSL3, INSL7, Relaxin-1, Testosterone, and MDA levels were measured in testicular tissue samples. INSL3, INSL7, and MDA levels were measured in liver tissue samples.

MDA was measured with colorimetrical method of Uchiyama and Mihara [39]. The principle of the MDA measurement method is based on the reaction of TBA (thiobarbutiric acide) and MDA in acidic pH. This method includes the sample that underwent alkaline hydrolysis, acid deproteinization, derivatization with TBA, and n-butanol extraction The wet weight of the tissue samples at pH 7.4 was measured, then divided into pieces and transferred into tubes and homogenized in 150 mM KCl at 4 °C to form 10% homogenate using a Misonix’s Microscan ultrasonic cell disruptor. The homogenate was added to 2 mL of 8% HClO_4_ and centrifuged at 3000 rpm for 15 min. Supernatants were obtained. A total of 0.5 mL of the supernatant, 3 mL of 1% H3PO4, and 1 mL of 0.675% TBA were mixed and incubated in a 90 °C water bath for 45 min. The MDA-TBA complex is extracted with 4 mL n-butanol which is used as a solvent to avoid interference formation by removing contaminants from incubation mixture. After cooling the phase in the tube, the butanol phase was separated by centrifugation, and absorbance was measured at 532 nm against n-butanol by Shimadzu UV-1601 (Japan) spectrometer. After the mixture tubes cooled down, the butanol phase occured. Then, the butanol phase was separated by centrifugation and absorbance measured at 532 nm against n-butanol blank by Shimadzu UV-1601 (Japan) spectrometer. Values were calculated as nmol/g tissue.

Calculation of MDA values:

$$\begin{array}{c}A=a\times b\times c\\ c=A/a\times b\\ \begin{array}{c}c=[A/1.56\times {10}^{5}{\mathrm{ cm}}^{-1}{{\text{M}}}^{-1}\times (1\mathrm{ cm})]\times \mathrm{dilution factor}\\ c=[A/1.56\times {10}^{5} {{\text{cm}}}^{-1}{{\text{M}}}^{-1}\times (1\mathrm{ cm})]\times \mathrm{dilution factor}\\ \begin{array}{c}c=A\times 108.9\mathrm{ nmol}/{\text{mL}}\\ c={\text{Xnmol}}/{\text{mL}}\end{array}\end{array}\end{array}$$

MDA values were determined as follows: *A* = *εlc*, where *ε* is the extinction coefficient of the MDA–TBA complex 1.56 × 10^5^ cm^–1^ M^–1^, *l* is the cell length (cm), and *c* is the concentration (M). By substituting, the concentration (in nmol/mL) is obtained from *c* = 108.9 × *A*. In order to calculate the MDA as nmol/g tissue, recorded tissue weights were divided by MDA concentration (nmol/mL) [39].

Commercial ELISA test kits were used for biochemical analyses. All the analysis was done according to kits procedures. INSL3 levels (ng/g tissue) with BT Lab ELISA test kit (Cat No: E2114Ra), INSL7 levels (ng/g tissue) with BT Lab ELISA test kit (Cat No: E1517RA), relaxin family peptide receptor 1 (RXFP1) levels (ng/g tissue) with BT Lab ELISA test kit (Cat No: E2544Ra), and testosterone levels (ng/g tissue) with BT Lab ELISA test kit (Cat No: EA0023Ra) were analyzed. Rayto microplate washer (RT-2600, China) was used as the Elisa washer, and BMG LABTECH (Germany) was used as the Elisa reader.

### Statistical Analysis

Biochemical data of the study were analyzed using statistical programme SPSS 22.0. Arithmetic means and standard deviations (mean ± SD) of all parameters were calculated. “Shapiro–Wilk” test was used to determine the homogeneity of the data, and it was determined that the data did not show normal distribution. The Kruskal–Wallis *H* test was used to determine the differences between the groups, and the Mann–Whitney *U* test was used to determine which group the difference originated from. Spearman correlation test was used for correlation analysis. Differences at p < 0.05 level were considered significant.

## Results

**Graph 1:**
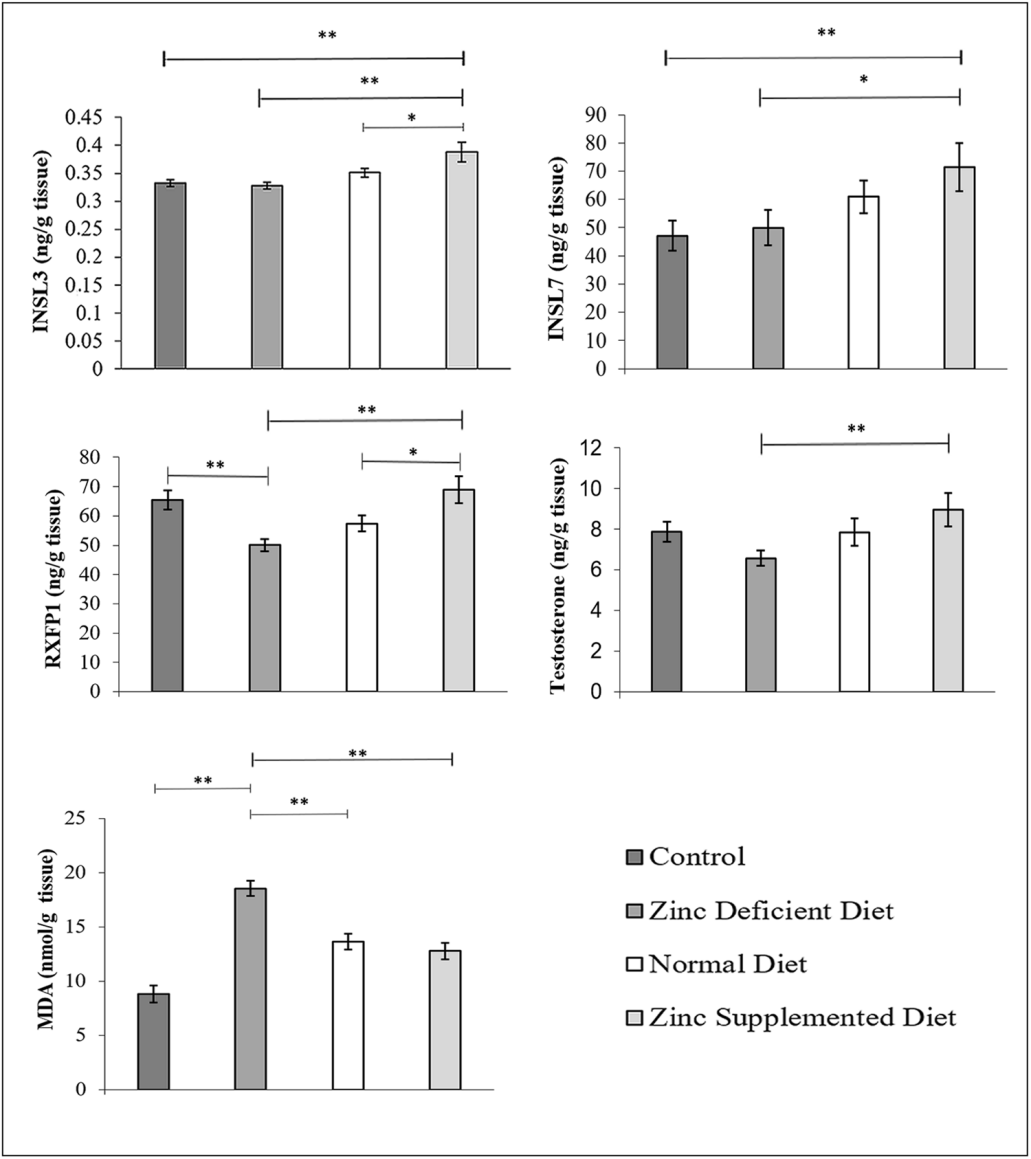
Comparison of INSL3, INSL7, RXFP1, testosterone, and MDA levels in testicular tissue of male offspring between the groups (mean ± SD)

Graph 1 shows the levels (mean ± SD) of various biochemicals in testicular tissues. INSL3 levels of the zinc-supplemented diet group (0.388 ± 0.05 ng/g tissue) were significantly (*p* < 0.05) higher than those of the control (0.333 ± 0.01 ng/g tissue), normal diet group (0.351 ± 0.02 ng/g tissue), and zinc-deficient diet group (0.328 ± 0.01 ng/g tissue).

INSL7 levels in the zinc-supplemented diet group (71.51 ± 26.84 ng/ g tissue) were higher than those in the control (47.11 ± 16.94 ng/ g tissue, *p* = 0.013) and the zinc-deficient diet group (50.00 ± 19.71 ng/g tissue, *p* = 0.027). The differences between the other groups were not significant.

RXFP1 levels in the zinc-supplemented diet group (68.99 ± 14.72 ng/g tissue) were significantly higher than those in the normal diet (57.38 ± 8.61 ng/g tissue, *p* = 0.018) and zinc-deficient diet (50.07 ± 6.65 ng/g tissue, *p* = 0.00) groups. RXFP1 levels in the control group (65.38 ± 10.28 ng/g tissue) were significantly (*p* = 0.002) higher than those in the zinc-deficient diet fed group.

Testosterone levels in the zinc-supplemented diet group (8.96 ± 2.60 ng/mL g tissue) were significantly higher than those in the zinc-deficient diet group (6.57 ± 1.19 ng/ g tissue). The levels were lower in the control group (7.88 ± 1.54 ng/g tissue) and in the group fed with a normal diet (7.85 ± 2.11 ng/g tissue). The differences between other groups were not significant.

MDA levels in the zinc-deficient diet group (18.54 ± 2.20 nmol/gr tissue) were significantly higher than those in the normal diet (13.66 ± 2.30 nmol/gr tissue, *p* = 0.00), zinc-supplemented diet (12.77 ± 2.47 nmol/gr tissue, *p* = 0.00), and control (8.83 ± 2.48 nmol/gr tissue, *p* = 0.00) groups. The lowest levels were found in the zinc-supplemented group.

As shown in Table 3, a positive correlation between INSL7 and INSL3 levels (*r* = 0.568, *p* = 0.000), a positive correlation between testosterone and RXFP1 (*r* = 0.355, *p* = 0.024), and a negative correlation between MDA and RXFP1 (*r* =  − 0.356, *p* = 0.024) were observed. Levels of INLS3 and INSL7 positively correlated (*r* = 0.721, *p* = 0.019) in the control group, whereas RXFP1 and INSL7 levels were negatively correlated (*r* =  − 0.685, *p* = 0.029). In the zinc-deficient group, INSL7 levels were negatively correlated with RXFP1 (*r* =  − 0.673, *p* = 0.033), whereas a positive correlation (*r* = 0.806, *p* = 0.005) was detected in the zinc-supplemented group. In the group fed with a normal diet, the correlation between INSL3 and INSL7 was positive (*r* = 0.646, *p* = 0.044).

**Table 3:** Correlation data of INSL3, INSL7, RXFP1, testosterone, and MDA levels in testicular tissue of male offsprings

Groups	INSL3 (ng/g tissue)		INSL7 (ng/ g tissue)		RXFP1 (ng/ g tissue)		Testosterone (ng/ g tissue)		MDA (nmol/g tissue)	
	*r*	*p*	*r*	*p*	*r*	*p*	*r*	*p*	*r*	*p*
*Total groups*										
INSL3 *n:40*	1	-	0.568**	**0.000**	0.206	0.202	0.134	0.411	− 0.021	0.9
INSL7 *n:40*	0.568**	**0.000**	1	-	− 0.053	0.746	0.099	0.545	− 0.006	0.969
RXFP1 *n:40*	0.206	0.202	− 0.053	0.746	1	-	0.355*	**0.024**	− 0.356*	**0.024**
Testosterone *n:40*	0.134	0.411	0.099	0.545	0.355*	**0.024**	1	-	− 0.245	0.128
MDA *n:40*	− 0.021	0.900	− 0.006	0.969	− 0.356*	**0.024**	− 0.245	0.128	1	-
*Control group*										
INSL3 *n:10*	1	-	0.721*	**0.019**	− 0.103	0.777	0.224	0.533	0.079	0.829
INSL7 *n:10*	0.721*	**0.019**	1	-	− 0.685*	**0.029**	0.394	0.260	− 0.297	0.405
RXFP1 *n:10*	− 0.103	0.777	− 0.685*	**0.029**	1	-	− 0.333	0.347	0.418	0.229
Testosterone *n:10*	0.224	0.533	0.394	0.260	− 0.333	0.347	1	-	0.321	0.365
MDA *n:10*	0.079	0.829	− 0.297	0.405	0.418	0.229	0.321	0.365	1	-
*Zinc-deficient diet group*										
INSL3 *n:10*	1	-	0.030	0.933	− 0.165	0.649	0.628	0.052	− 0.474	0.166
INSL7 *n:10*	0.030	0.933	1	-	− 0.673*	**0.033**	− 0.067	0.855	− 0.219	0.544
RXFP1 *n:10*	− 0.165	0.649	− 0.673*	**0.033**	1	-	− 0.139	0.701	− 0.377	0.283
Testosterone *n:10*	0.628	0.052	− 0.067	0.855	− 0.139	0.701	1	-	− 0.164	0.650
MDA *n:10*	− 0.474	0.166	0.219	0.544	− 0.377	0.283	− 0.164	0.650	1	-
*Normal diet group*										
INSL3 *n:10*	1	-	0.646*	**0.044**	− 0.234	0.515	− 0.178	0.622	0.554	0.097
INSL7 *n:10*	0.646*	**0.044**	1	-	− 0.588	0.074	− 0.079	0.829	− 0.067	0.855
RXFP1 *n:10*	− 0.234	0.515	− 0.588	0.074	1	-	0.455	0.187	0.212	0.556
Testosterone *n:10*	− 0.178	0.622	− 0.079	0.829	0.455	0.187	1	-	0.224	0.533
MDA *n:10*	0.554	0.097	− 0.067	0.855	0.212	0.556	0.224	0.533	1	-
*Zinc-supplemented diet group*										
INSL3 *n:10*	1	-	0.529	0.116	0.565	0.089	− 0.055	0.881	0.006	0.987
INSL7 *n:10*	0.529	0.116	1	-	0.806**	**0.005**	0.261	0.467	− 0.079	0.829
RXFP1 *n:10*	0.565	0.089	0.806**	**0.005**	1	-	0.103	0.777	0.309	0.385
Testosterone *n:10*	− 0.055	0.881	0.261	0.467	0.103	0.777	1	-	− 0.333	0.347
MDA *n:10*	0.006	0.987	− 0.079	0.829	0.309	0.385	− 0.333	0.347	1	-

Spearman correlation analysis was considered significant at *p < 0.05, **p < 0.01

**Graph 2:**
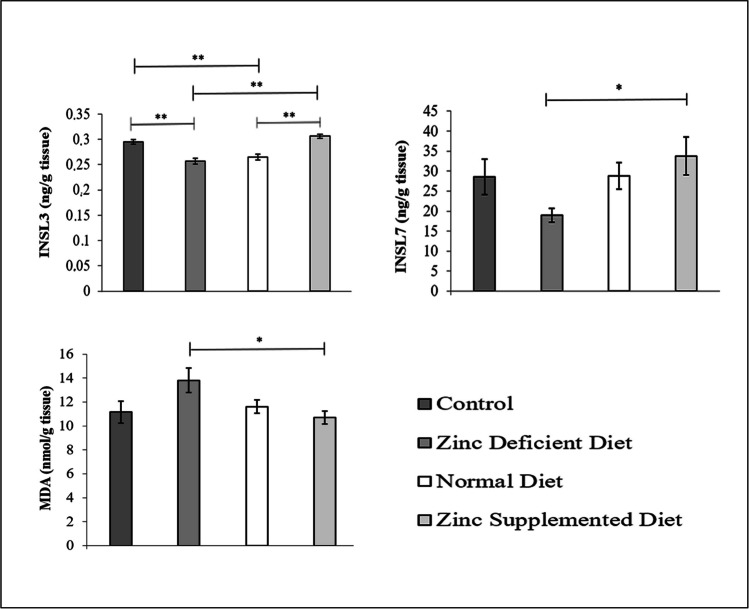
Comparison of INSL3, INSL7, and MDA levels in liver tissue of male offspring between groups (mean ± SD)

As shown in Graph 2, INSL3 levels in the zinc-supplemented diet group (0.306 ± 0.01 ng/g tissue) were significantly higher (*p* = 0.00) than those in the normal diet group (0.265 ± 0.01 ng/g tissue) and in the zinc-deficient diet group (0.257 ± 0.01 ng/g tissue). The difference between the zinc-supplemented diet group and the control group (0.295 ± 0.01 ng/g tissue) was not significant (*p* = 0.424).

INSL7 levels in the zinc-supplemented diet group (33.78 ± 14.98 ng/g tissue) were significantly higher (*p* = 0.039) than those in the zinc-deficient diet group (18.96 ± 5.48 ng/g tissue). INSL7 levels were 28.56 ± 13.98 ng/g tissue in the control group and 28.79 ± 10.49 ng/g tissue in fed with normal diet group.

MDA levels in the zinc-deficient diet group (13.81 ± 3.27 nmol/gr tissue) were significantly higher than those in the zinc-supplemented diet group (10.71 ± 1.67 nmol/gr tissue, *p* = 0.041). The differences (the normal diet group and control group) were not significant. The group fed with a normal diet (11.16 ± 2.86 nmol/g tissue) had levels like those of the control group (11.62 ± 1.80 nmol/g tissue). The lowest levels were found in the zinc-supplemented diet group.

As shown in Table 4, a positive (*r* = 0.568, *p* = 0.00) correlation was found between INSL7 and INSL3 levels in the total group. According to the correlation data of the control group and the normal diet group, a positive correlation (*r* = 0.767, *p* = 0.010; *r* = 0.638, *p* = 0.047) was detected between INSL3 and INSL7, respectively. In the group fed with zinc-supplemented group, a positive correlation was determined between INLS3 and INSL7 (*r* = 0.806, *p* = 0.005).

**Table 4:** Correlation data of INSL3, INSL7, and MDA levels in liver tissue of male offspring

Groups	INSL3 (ng/g tissue)		INSL7 (ng/g tissue)		MDA (nmol/g tissue)	
	*r*	*p*	*r*	*p*	*r*	*p*
*Total group*						
INSL3 *n:40*	1	-	0.568**	**0.000**	− 0.131	0.420
INSL7 *n:40*	0.568**	**0.000**	1	-	− 0.016	0.922
MDA *n:40*	0.131	0.420	− 0.016	0.922	1	-
*Control group*						
INSL3 *n:10*	1	-	0.767*	**0.010**	0.382	0.276
INSL7 *n:10*	0.767*	**0.010**	1	-	− 0.091	0.803
MDA *n:10*	0.382	0.276	0.091	0.803	1	-
*Zinc-deficient diet group*						
INSL3 *n:10*	1	-	0.576	0.082	− 0.140	0.700
INSL7 *n:10*	0.576	0.082	1	-	− 0.345	0.328
MDA *n:10*	− 0.140	0.700	− 0.345	0.328	1	-
*Normal diet group*						
INSL3 *n:10*	1	-	0.638*	**0.047**	0.215	0.551
INSL7 *n:10*	0.638*	**0.047**	1	-	− 0.006	0.987
MDA *n:10*	0.215	0.551	− 0.006	0.987	1	-
*Zinc-supplemented diet group*						
INSL3 *n:10*	1	-	0.806*	**0.005**	0.067	0.855
INSL7 *n:10*	0.806*	**0.005**	1	-	0.273	0.446
MDA *n:10*	0.067	0.855	0.273	0.446	1	-

Spearman correlation analysis was considered significant at *p < 0.05, **p < 0.01

## Discussion

This study is the first to investigate how zinc sulfate affects the molecular pathways that regulate hormones such as INSL3, INSL7, RXFP1, and testosterone in the prepubertal and pubertal periods. These proteins play important roles in spermatogenesis and fertilization.

### Evaluation of the Biochemical Results of Testis

Joshi et al. conducted a study on 60 prepubertal rats separated into control, zinc-supplemented group, and zinc deficient diet 4 groups for 4 weeks. They reported that zinc deficiency in the prepubertal period disrupted the prostate structure, increased 3β-hydroxysteroid dehydrogenase activity and nitric oxide levels, and decreased total protein concentration [40]. However, our study showed that zinc-supplemented diets enhanced spermatogenesis in rats separated from their mothers. Levels of INSL3, RXFP1, and testosterone in testicular tissue of the zinc-deficient diet group were lower compared with those of the zinc-supplemented diet group (*p* < 0.05). INSL7 levels significantly differed between groups 1, 2, and 4.

INSL3 is a fetal hormone that affects maternal and placental pathology and physiology. It also promotes in the development of the male reproductive organ, particularly in testicular descent during embryonic development [41]. Experimental studies have shown that cryptorchidism is associated with genetic abnormalities occurring in INSL3/RXFP2. Allelic variants in the INSL3 and RXFP2 genes have only been found in patients with undescended testes [42–44].

Bogatcheva et al. conducted *in vitro* experiments and concluded that the T222P mutation of RXFP2, an INSL3 receptor, prevented the expression of the receptor on the cell surface, hindering testicular descent [44]. Overall, the phenotypes of cryptorchid males with mutations in the INSL3 and RXFP2 genes vary significantly from unilateral cryptorchidism in the first year of life to persistent bilateral cryptorchidism [43, 45]. In our study, INSL3 was significantly lower in the zinc-deficient diet group, suggesting that zinc supplementation would benefit offspring from maternal zinc-deficient diets.

It is well known that INSL3 is required for the development of wolf ducts and masculinization of the gubernaculum [46]. Accordingly, when we compared groups in our study, zinc supplementation increased INLS3 levels in both normal diet fed mothers and normal diet-fed offspring.

Ferlin and Foresta [47] measured INSL3 levels in healthy and pathological male testes and found that most circulating INSL3 in adult males is of testicular origin. Negligible INSL3 production was measured in subjects with excessive testicular damage, and INSL3 levels decreased in infertile men with hypospermatogenesis. The concentration of INSL3 hormone reportedly reflects the functional status of Leydig cells [47]. Although the release of INSL3 is maximal in the prepubertal period, it persists throughout life and is important for diagnosing and assessing the reproductive quality of individuals.

Furthermore, Huang et al. [48] found that deletion of INSL3 or RXFP2 in mice causes cryptorchidism due to failure of gubernaculum development. However, RXFP2 deletion in gubernacular smooth or striated muscle cells did not compromise testicular descent or development. Specific ablation of RXFP2 in male germ cells using the stra8-icre (stimulatory retinoic acid gene 8) transgene did not affect testicular descent, spermatogenesis, or fertility in adult males. In addition, no significant changes in germ cell apoptosis were detected in mutant males. They reported that INSL3/RXFP2 signaling is important for testicular descent but abandonable for spermatogenesis and fertility in adult males [48]. Serum INSL3 levels are associated with increasing age, onset of puberty, and testicular volume and regulated by testosterone and INSL3 [49]. However, we did not find a significant correlation between testosterone and INSL3 levels. The relationship between INLS3 and testosterone may be indirectly mediated by sex hormone-binding proteins. Future studies should elucidate the relationship between INSL3 and testosterone by analyzing sex hormone-binding proteins.

Ali et al. [50] measured zinc levels in fertile and infertile populations to establish a relationship with testosterone. Testosterone levels were significantly lower in the oligospermic and azoospermic groups than in the normospermic and control groups. Similarly, seminal zinc levels were reported to be low in azoospermic and oligospermic groups. A possible role for zinc in spermatogenesis and steroidogenesis has been suggested. Based on the low zinc concentration found in infertile individuals, they reported that zinc is responsible for testicular function and that zinc plays a role in spermatogenesis and steroidogenesis [50]. In our study, testosterone levels in the testes of the zinc-supplemented diet group were significantly higher than those of the zinc-deficient diet group, therefore, which agrees with previous findings.

Symptoms of late-onset hypogonadism vary and can not be only express by determining the circulation level of testosterone. Four different hormones (testosterone, AMH, INSL3, and InhB) are secreted into the testes. Chong et al. [51] have shown that these hormones change independently in young (19–50 years) and old (70–90 years) men and are influenced by the number of endocrine cells. As levels of testicular hormones in older men vary, the testis may age differently across men. Another study found that age-related decreases in testosterone, and INSL3 levels were related to each other [52].

The RXFP1 receptor has agonist affinity for INSL7 [53]. RXFP1 activates pleiotropic signaling pathways including the signalosome protein complex [54]. Yang et al. [55] identified two INSL7 genes in the testes, INSL7a and INSL7b, which are predominantly expressed in the testis and brain, respectively. To understand the role of INSL7 in testis development, a homologous null- INSL7 gene mutant line was generated using CRISPR/Cas9 technology. They detected significant decreases in spermatogonia, spermatocytes, spermatids, and spermatogenic cells in spermatozoa with a mutation inINSL7a. It has also been reported to suppress the expression of genes that affect germ cells and Leydig cells, increase stillbirths, deform sperm, and decrease sperm motility. INSL7 has been shown to mediate androgens in the testis via the HPG axis [55]. In our study, INSL7, which is localized in testicular tissues, was found to be significantly higher in the testicular tissues of the zinc-supplemented diet group than in the zinc-deficient diet group. A maternal zinc-deficient diet did not significantly affect INSL7 levels in the offsprings. However, a zinc-supplemented diet altered INLS7 levels in the offspring. Therefore, the diet of the offspring had a greater effect on INSL7 levels. Filonzi et al. [56] investigated the function of relaxin in the vas deferens, a tissue with high expression of the INSL7 receptor RXFP1 in the reproductive duct of the male rat. RXFP1 is found in almost all parts of the male reproductive system, especially the testis and vas deferens. RXFP1 has also been detected in Sertoli cells, which may be important in spermatogenesis. Although relaxin did not affect contractility in the vas deferens, it may affect vascular compliance and remodeling of the collagen matrix [56]. Pimenta et al. [57] investigated the role of INSL7 in spermatogenesis and found that INSL7 and RXFP1 may be indirectly stimulate Sertoli cells or directly affect germ cells. Relaxin may increase the number of meiotic cells [57]. Here, RXFP1 levels were highest in the zinc-supplemented group, which was higher than all other groups. In addition, zinc affected RXFP1 levels even with feeding with standard diet and feeding zinc deficient diet to both mother and offspring decreased RXFP1 levels. Intragroup correlations between INSL7 and RXFP1 were negative in the zinc-deficient group and positive in the zinc-supplemented group, suggesting that zinc mediates the relationship between INSL7 and its receptor. Zinc deficiency may affect the affinity of INSL7 for its receptor.

MDA is a key biomarker to lipid peroxidation. Increased free radical and oxidant formation is thought to accelerate testicular damage [58]. Oteiza et al. [59] found that zinc deficiency damaged Leydig cells, decreased steroid synthesis and testicular growth, and increased oxidative stress in rats fed a zinc-deficient diet for 7 days [59]. Omu et al. [60] investigated the mechanisms behind zinc deficiency on spermatogenesis in rats. Rats were divided into three groups: zinc supplemented, zinc-deficient, and control groups. After 4 weeks, decreased testosterone production, increased MDA levels, and impaired spermatogenesis were observed in the zinc-deficient diet group [60]. Our study also found decreased testosterone levels and increased MDA levels in the zinc-deficient group. Therefore, zinc supplementation can reduce lipid peroxidation which is crucial for sexual development in the prepubertal and pubertal periods. In our study, testosterone was positively correlated with RXFP1 and MDA was negatively correlated with RXFP1, suggesting that testosterone is effective and that lipid peroxidation and the number of receptors are inversely related.

In light of our findings, zinc may increase testosterone synthesis in Leydig cells by eliminating free radicals, and zinc supplementation may increase spermatogenesis in rats with normal reproductive function. A limitation of our study is that we did not measure zinc in testicles. Therefore, this study is preliminary. To fully determine the relationship between zinc and the relaxin family, metabolic changes should be assessed in the testicle following zinc supplementation.

### Evaluation of Biochemical Results of the Liver

This study is also the first to investigate how zinc sulfate affects pathways that regulate of hormonal factors such as INSL3 and INSL7 that affect liver function in prepubertal and pubertal periods. However, we explored how zinc enhances these protein structures.

As a critical organ, the liver [61] regulates zinc homeostasis. Zinc is required for proper liver function [62, 63]. Severe maternal zinc deficiency results in growth retardation and high mortality during embryonic development in humans [64]. Irregular intake/ or deficiency of zinc contributes to acute and chronic liver disease. Numerous clinical studies have shown that zinc supplementation can be used to not only prevent but also treat disease [65]. Zinc supplementation provides protection in experimental animal models of acute and chronic liver injury; however, these hepatoprotective properties have not been fully elucidated.

In a study investigating the effect of INSL7 and INSL3 treatment on the cirrhotic liver, Bennett et al. [65] proposed that relaxin exerts antifibrotic effects on hepatic stellate cells (HSC) and deposits collagen in cirrhosis. Relaxin3 protects organs from excessive extracellular matrix accumulation and hepatic fibrosis, which commonly occur with aging. Collagen accumulation and HSC activation decreased in relaxin3-induced hepatic fibrosis and may be effective in the treatment of hepatic fibrosis, although hepatic damage persists [66].

Although several studies have measured INSL3 and INSL7 levels in liver tissue, what distinguishes our study is the evaluation of various zinc diets fed to male offspring of mothers fed a zinc-deficient diet.

Here, INSL3 levels were significantly higher in the zinc-supplemented diet group than in the normal diet and zinc-deficient diet groups (*p* < 0.05), whereas there was no significant difference from the control group. Thus, INSL3 increased with zinc concentration and zinc levels in offspring of mothers fed a zinc-deficient diet reached the values of the healthy control group after zinc supplementation. This is supported by the fact that INSL3 levels in offspring fed with a normal diet and born from zinc-deficient mothers did not reach levels of the healthy control. Therefore, zinc supplements may enhance INSL3 function in the liver.

The highest and lowest INSL7 levels were found in the zinc-supplemented and zinc-deficient groups, respectively. Although the difference between these groups was statistically significant, the INSL3 values of the zinc supplemented group exceeded the values of the healthy control group. Therefore, INSL7 levels reached normal levels with zinc supplementation.

Zinc is believed to play an important role in oxidant formation in liver tissue [67]. Here, the highest level of MDA was found in the group fed a zinc-deficient diet and the difference between this group and the zinc-supplemented group was significant. A positive correlation was found between INSL3 and INSL7 in the zinc-supplemented diet group. Ultimately, zinc supplementation may promote liver function given its ability to eliminate free radicals. However, further studies are needed to be able to confirm this.

Adequate dietary zinc bolsters the antioxidant defense system; zinc deficiency is associated with an increased risk of oxidative damage. Indeed, studies have shown a relationship between lipid, protein, DNA oxidation, and zinc deficiency [68–72]. Uddin et al. measured MDA and Zn levels in chronic liver disease (CLD) patients and healthy volunteers. High MDA levels were found in patients, with CLD, whereas zinc levels were lower than in the control group [73]. Here, MDA levels increased in the zinc-deficient diet group. Therefore, zinc supplementation may reduce lipid peroxidation and enhance the development of liver tissue in the prepubertal and pubertal periods.

## Conclusion

INSL3, INSL7, and RXFP1 are newly discovered and important.

Biomarkers whose roles in gonadal development remain unclear. Zinc-supplemented diets regulate in INSL3, INSL7, RXFP1, and testosterone levels during spermatogenesis. Moreover, zinc supplementation may enhance the functions of relaxin family members which are reportedly involved in puberty.

Zinc deficiency during embryonic development contributes to the pathogenesis of both acute and chronic liver diseases. Proliferative changes in the fetus have important consequences in the adult period. However, zinc-supplemented diets can alter INSL3 and INSL7 levels in liver tissue during the prepubertal/pubertal period.

Future studies are needed to investigate the biological structures behind these mechanisms that may cause infertility, to determine the distribution of these hormones, and to explore how zinc affects hormones secreted from the testicular tissue.

## Data Availability

The datasets generated during and/or analyzed the current study are available on reasonable request.
